# Is patient-grouping on basis of condition on admission indicative for discharge destination in geriatric stroke patients after rehabilitation in skilled nursing facilities? The results of a cluster analysis

**DOI:** 10.1186/1472-6963-12-443

**Published:** 2012-12-04

**Authors:** Bianca I Buijck, Sytse U Zuidema, Monica Spruit-van Eijk, Hans Bor, Debby L Gerritsen, Raymond TCM Koopmans

**Affiliations:** 1 Department of Primary and Community Care, Centre for Family Medicine, Geriatric Care and Public Health, Radboud University Nijmegen Medical Centre, P.O.Box 9101, Nijmegen, 6500 HB, the Netherlands; 2 De Zorgboog, Roessel 3, Bakel, 5761 RP, the Netherlands; 3 SVRZ, Koudekerkseweg 143, Middelburg, 4335 SM, the Netherlands; 4 Department of General Practice, University of Groningen, University Medical Center Groningen, P.O.Box 30001, Groningen, 9700 RB, the Netherlands

**Keywords:** Stroke, Geriatric, Rehabilitation, Skilled-nursing-facility

## Abstract

**Background:**

Geriatric stroke patients are generally frail, have an advanced age and co-morbidity. It is yet unclear whether specific groups of patients might benefit differently from structured multidisciplinary rehabilitation programs. Therefore, the aims of our study are 1) to determine relevant patient characteristics to distinguish groups of patients based on their admission scores in skilled nursing facilities (SNFs), and (2) to study the course of these particular patient-groups in relation to their discharge destination.

**Methods:**

This is a longitudinal, multicenter, observational study. We collected data on patient characteristics, balance, walking ability, arm function, co-morbidity, activities of daily living (ADL), neuropsychiatric symptoms, and depressive complaints of 127 geriatric stroke patients admitted to skilled nursing facilities with specific units for geriatric rehabilitation after stroke.

**Results:**

Cluster analyses revealed two groups: cluster 1 included patients in poor condition upon admission (n = 52), and cluster 2 included patients in fair/good condition upon admission (n = 75). Patients in both groups improved in balance, walking abilities, and arm function. Patients in cluster 1 also improved in ADL. Depressive complaints decreased significantly in patients in cluster 1 who were discharged to an independent- or assisted-living situation. Compared to 80% of the patients in cluster 2, a lower proportion (46%) of the patients in cluster 1 were discharged to an independent- or assisted-living situation.

**Conclusion:**

Stroke patients referred for rehabilitation to SNFs could be clustered on the basis of their condition upon admission. Although patients in poor condition on admission were more likely to be referred to a facility for long-term care, this was certainly not the case in all patients. Almost half of them could be discharged to an independent or assisted living situation, which implied that also in patients in poor condition on admission, discharge to an independent or assisted living situation was an attainable goal. It is important to put substantial effort into the rehabilitation of patients in poor condition at admission.

## Background

In the past decade, there has been increasing interest in stroke rehabilitation. In 2004, 15 million people suffered a stroke worldwide [1]. The expectation is that the number of patients with stroke will rise in the future, because of the ageing of the population; accordingly, there will be a growing demand for rehabilitation services. In the Netherlands, the incidence of stroke is expected to rise from 1.8 per 1,000 persons in the year 2000 to 2.8 per 1,000 persons in 2020 [2].

More than a quarter of all patients die after a stroke within one to three months [3]. In the Netherlands if the patient survives after the acute phase in the hospital, a stroke patient is referred to either rehabilitation centers or specific geriatric rehabilitation units in skilled nursing facilities (SNFs). Referral depends on the patients’ age, general condition and level of impairment. Dutch SNFs provide elderly patients after a stroke with low-intensity multidisciplinary rehabilitation programs, with the objective to discharge them to an independent-living situation. Patients receiving rehabilitation in these SNFs are generally frail, have an advanced age and are suffering from co-morbidity. Therefore, the more demanding rehabilitation in rehabilitation centers is not appropriate for these elderly patients [4].

Rehabilitation in SNFs is provided by a multidisciplinary team consisting of an elderly care physician [5], a physiotherapist, an occupational therapist, a speech-language therapist, a psychologist, a dietician, and nursing staff. The overall amount of multidisciplinary treatment in SNFs [6] is approximately 4 hours per week per patient, evenly distributed over 5 working days. Therapy sessions in SNFs consist of a combination of individual and group therapies, aimed at restoring (functional) abilities. Based on their clinical judgment of the condition at admission, therapists decide on the content of their treatment and tailor the treatment to the needs of the patient. However, rehabilitation could be more efficient if it were possible to refer newly admitted patients to standardized rehabilitation programs with various levels of intensity. Research showed that patients receiving a program of focused stroke rehabilitation performed better than other patients [7].

In literature, age and disability upon admission are the most important determinants of rehabilitation outcome after stroke [7-11]. However, regardless of the degree of physical impairment at baseline, during the rehabilitation process, which takes from a few weeks to 1 year, several additional factors such as comorbidity [12], therapy intensity [13], early start of therapy following stroke [13,14], motivation of the patient, support of relatives, neuropsychiatric symptoms [15], and environment [13] can influence rehabilitation outcomes and discharge destination. Therefore, in order to identify meaningful patient groups for developing specific rehabilitation programs in SNFs, the aims of our study are 1) to determine relevant patient characteristics to distinguish groups of patients based on their admission scores in SNFs, and (2) to study the course of these particular patient-groups in relation to their discharge destination.

## Methods

### Design

This study is part of the Nijmegen Geriatric Rehabilitation in AMPutation and Stroke study (GRAMPS), which is a longitudinal, multicenter, observational study of geriatric patients admitted to SNFs for rehabilitation. Data were collected from January 2008 until January 2009 in 15 Dutch SNFs, all of which are part of the Nijmegen University Nursing Home Network (UKON: http://www.uko-n.nl) of the Radboud University Nijmegen Medical Center. All 15 of the SNFs are situated in the southern part of the Netherlands. Data were collected upon admission to the SNF and at discharge to an independent/assisted-living situation or at referral to long-term care in a nursing home. The medical ethical committee of the region Nijmegen-Arnhem approved the research protocol of the GRAMPS study. The research protocol of the GRAMPS stroke-study has been extensively described elsewhere [16].

### Patients

All patients admitted to the 15 SNFs for rehabilitation after stroke were asked to participate. Four categories of patients were excluded from participation: 1) patients who declined informed consent, 2) patients who were legally incapable to give informed consent, 3) patients who were expected to be discharged within 2 weeks, 4) critically ill patients.

### Measurements

From the medical records the following patients’ characteristics were collected: age, gender, first stroke or recurrent stroke, stroke subtype (ischemic/non-ischemic) and localization of stroke.

Information about comorbidity was registered using the Charlson Index (CI). The CI comprises 19 categories of diagnoses from the International Classification of Diseases (9th revision Clinical Modification ICD-9CM) [17]. We used the adjusted CI, because two items in the original version (“cerebrovascular disease” and “hemiplegia”) reflect the stroke itself rather than additional morbidity. The adjusted CI is validated in clinical stroke outcome studies [18]. The CI scores were summed.

We used the Barthel Index (BI), modified by Collin et al. in 1988 [19,20] for measuring dependency in activities of daily living (ADL). The total score ranges from 0–20. A score of 20 represents complete functional independence.

The Frenchay Arm Test (FAT) was used to evaluate arm function after stroke. The patient is asked to perform five activities with the affected arm, and he or she awards one point for each successfully completed activity [21].

The patients balance was assessed using the Berg Balance Scale (BBS). This is an ordinal 14-item scale (0–56 points) developed by Berg et al. [22,23].

The FAC score assesses a patient’s ability to walk independently of other people. It has an ordinal six point scale. Zero indicates total dependency and five indicates fully independent walking. The use of a walking device is permitted during the test [24].

To assess neuropsychiatric symptoms, the Neuropsychiatric Inventory: Nursing Home (NPI-NH) version, which is applicable in various patient groups, was used [25-27]. The NPI comprises 12 symptoms: delusions, hallucinations, agitation/aggression, depression, anxiety, euphoria, disinhibition, irritability/lability, apathy, aberrant motor behavior, sleeping disturbances, and eating changes. Symptoms within each domain are rated by the nurse in terms of both frequency (1 to 4) and severity (1 to 3), yielding a composite symptom score (frequency x severity). The 12 composite symptom scores were summed to obtain an NPI total score.

The eight-item version of the Geriatric Depression Scale (GDS-8) was used to screen for depressive complaints. It is a shortened patient-friendly test derived from the GDS-15 version and is developed specifically for the nursing home population [28,29].

All of the measurements described above were performed within 3 weeks after admission to the SNF. Measures of BI, FAT, BBS, FAC, NPI-NH, and GDS-8 were repeated in the two weeks before discharge.

### Statistical analysis

To identify meaningful groups of patients, we first performed a Two-step Cluster Analysis to identify variables that discriminate between groups. Cluster analysis aims to create groups in which the degree of association between objects is maximal if they belong to the same group and minimal otherwise.

We entered age, gender, information about the stroke (stroke type, localization, first stroke) and measurements on admission of CI, BI, FAT, BBS, FAC, NPI-NH, and GDS-8 in the two-step cluster analysis. Data about cognition, aphasia and swallowing [16] on admission were also entered in the cluster model, but appeared to be not statistically significant and were left out of the final analysis. In the next step, we determined whether patients assigned to each cluster had a different rehabilitation outcome in terms of the percentage of successful rehabilitation (discharge to independent/assisted-living situation within 1 year after admission), change in functional outcomes (BI, FAT, BBS, and FAC), change in neuropsychiatric symptoms (NPI-NH), and depressive complaints (GDS-8) during the rehabilitation. Differences between the two clusters were evaluated using the Mann–Whitney-*U* Test and the Chi-squared Test. Changes with respect to the baseline scores and the scores at the end of rehabilitation were tested using the Wilcoxon Signed-Rank Test. The Kruskall-Wallis Test was used to test for differences between groups with reference to the changes between baseline and end of rehabilitation scores. All data were processed using SPSS 18 [30].

## Results

Of the 378 eligible patients, 186 were included in the GRAMPS study. Patients were excluded from the study based on unwillingness to give informed consent (*n* = 73), critical illness (*n* = 13), legal incapacity (*n* = 64), and expected short stay (*n* = 7). In addition, 35 patients were not asked to participate for logistic reasons; e.g. during holidays, every second patient was included to prevent overburdening of the personnel. The excluded patients did not significantly differ from those included in the study in terms of age, gender, or length of stay in the SNF. Patients were admitted to the hospital at day one of the stroke, and stayed a mean 23 days in hospital after stroke (range 9 days - 6 weeks).

In the present study, a complete data set was obtained of 127 patients, these patients were included in the cluster analysis. The admission scores (variables in the cluster analysis) of patients with incomplete data were not different from those included in the analyses, accept for age (mean age 76 and 80 years for excluded and included patients respectively; *p* < 0.05).

Table 1 shows the results of the cluster analysis, based on admission data. Patients appeared to cluster in two groups. The groups can be meaningfully described as a cluster with patients in poor condition on admission (cluster 1, “poor cluster”) and a cluster with patients in fair/good condition on admission (cluster 2, “good cluster”).

**Table 1:** Description sample and clusters

**Variable**	**Total sample**	**%**	**Cluster 1 (poor)**	**%**	**Cluster 2 (good)**	**%**	**Significant difference between clusters**
	**Median (IQR)**		**Median (IQR)**		**Median (IQR)**		
	**n = 127**		**n = 52**		**n = 75**		
Berg Balance Scale (range 0–56)	31 (5, 46)		3 (0, 17)		44 (33, 50)		**
Functional Ambulation Categories (range 0–5)	3 (0, 4)		0 (0, 2)		4 (3, 4)		**
Barthel Index (range 0–20)	12 (6, 16)		5 (2, 9)		14 (13, 18)		**
Frenchay Arm Test (range 0–5)	4 (1, 5)		1 (0, 4)		5 (4, 5)		**
Charlson Index (range 0–27)	2 (1, 3)		3 (2, 3)		2 (1, 2)		**
Global Depression Scale (range 0–8)	1 (0, 3)		2 (1, 4)		1 (0, 2)		**
Neuropsychiatric Inventory: Nursing Home version (range 0–144)	0 (0, 5)		4 (0, 12)		0 (0, 2)		**
Gender: male		48		35		57	*
Localization of stroke on right		58		65		54	
Ischemic stroke		85		90		81	
First stroke		83		87		80	
Age (years)	80 (76, 85)		81 (76, 86)		79 (76, 85)		

Cluster 2 *n* = 75 (59.1% of the patients).

Cluster 1 *n* = 52 (40.9% of the patients).

Asterisks indicate significant differences between groups Mann–Whitney-*U* Test and Chi-squared Test (**p < 0.01, *p < 0.05).

Variables that best separate are at the top of the table and descending.

Table 2 shows the change in scores between admission and discharge of the two clusters. Patients in the poor cluster significantly improved in ADL (BI), balance (BBS), walking ability (FAC) and arm function (FAT). Patients in the good cluster significantly improved in ADL (BI), balance (BBS) and walking ability (FAC).

**Table 2:** Changes in scores between admission and discharge

**Variables**		**Cluster 1 (poor)**		**Cluster 2 (good)**		**Cluster 1 (poor)**		**Cluster 1 (poor)**		**Cluster 2 (good)**		**Cluster 2 (good)**
		**median (IQR)**		**median (IQR)**		**Discharged median (IQR)**		**Long Term Care median (IQR)**		**Discharged median (IQR)**		**Long Term Care median (IQR)**
**BI admission (ADL)**	n = 52	5 (2, 9)	n = 75	14 (13, 18)	n = 24	6 (3, 10)	n = 28	4 (2, 7)	n = 60	15 (13, 18)	n = 15	13 (11, 17)
**BI discharge**	n = 41	11 (6, 15)**	n = 70	18 (16, 20)**	n = 23	14 (13, 16)**	n = 18	5 (3, 9)	n = 60	19 (17, 20)**	n = 10	10 (5, 13)
**BBS admission (balance)**	n = 52	3 (0, 17)	n = 75	44 (33, 50)	n = 24	9 (1, 25)	n = 28	2 (0, 4)	n = 60	44 (35, 50)	n = 15	35 (27, 47)
**BBS discharge**	n = 39	28 (3, 39)**	n = 66	48 (41, 52)**	n = 22	38 (28, 45)**	n = 17	3 (0, 15)*	n = 56	49 (44, 52)**	n = 10	26 (17, 53)
**FAC admission (walking ability)**	n = 52	0 (0, 2)	n = 75	4 (3, 4)	n = 24	0 (0, 2)	n = 28	0 (0, 1)	n = 60	4 (3, 4)	n = 15	3 (3, 4)
**FAC discharge**	n = 39	3 (0, 4)**	n = 69	5 (4, 5)**	n = 22	4 (3, 4)**	n = 17	0 (0, 2)	n = 58	5 (4, 5)**	n = 11	3 (3, 5)
**FAT admission (arm function)**	n = 52	1 (0, 4)	n = 75	5 (4, 5)	n = 24	1 (0, 3)	n = 28	1 (0, 5)	n = 60	5 (4, 5)	n = 15	5 (4, 5)
**FAT discharge**	n = 38	1 (0, 5)*	n = 65	5 (5, 5)	n = 21	3 (0, 5)**	n = 17	0 (0, 4)	n = 57	5 (5, 5)*	n = 8	5 (1, 5)
**GDS-8 admission (depressive complaints)**	n = 52	2 (1, 4)	n = 75	1 (0, 2)	n = 24	1 (0, 4)	n = 28	2 (1, 5)	n = 60	0 (0, 1)	n = 15	1 (0, 2)
**GDS-8 discharge**	n = 35	1 (0, 2)	n = 61	0 (0, 1)	n = 20	1 (0, 2)**	n = 15	1 (0, 4)	n = 54	0 (0, 0)	n = 7	4 (1, 5)
**NPI-NH admission (neuropsychiatric symptoms)**	n = 52	4 (0, 12)	n = 75	0 (0, 2)	n = 24	2 (0, 8)	n = 28	7 (0, 18)	n = 60	0 (0, 1)	n = 15	0 (0, 4)
**NPI-NH discharge**	n = 40	0 (0, 6)	n = 70	0 (0, 1)	n = 22	0 (0, 1)**	n = 18	4 (0, 15)	n = 60	0 (0, 0)	n = 10	2 (0, 7)

Asterisks indicate significant changes between admission and discharge within the four groups, Wilcoxon Signed- Rank Test (**p < 0.01, *p < 0.05).

*BI*: Barthel Index (range 0–20).

*BBS*: Berg balance Scale (range 0–56).

*FAC*: Functional Ambulation Categories (range 0–5).

*FAT*: Frenchay Activities Index (range 0–5).

*GDS-8*: geriatric Depression Scale (range 0–8).

*NPI-NH*: NeuroPsychiatric Inventory Nursing Home version (range 0–144).

A total of 84 patients (66%) were discharged to an independent/assisted-living situation, and 43 (34%) were referred for long-term care to a nursing home. Within the poor cluster (*n* = 52), 28 patients (54%) were referred to a nursing home for long-term care after rehabilitation and 24 patients (46%) were discharged to an independent/assisted-living situation. In the good cluster (*n* = 75), 15 patients (20%) were referred for long-term care to a nursing home and 60 patients (80%) were discharged to an independent/assisted-living situation. The percentage of discharge to an independent/assisted-living situation differed significantly between the good and the poor cluster (*p* < 0.0005).

Patients in the poor cluster who were discharged showed improvement of ADL (BI), balance (BBS), arm function (FAT), walking ability (FAC) and had a decrease of depressive complaints (GDS-8) and neuropsychiatric symptoms (NPI). The discharge scores of patients in the poor cluster who were discharged to an independent/assisted-living situation were almost similar to the on admission scores of the patients in the good cluster who were discharged. Patients in the good cluster who were discharged showed improvement in ADL (BI), balance (BBS), arm function (FAT) and walking ability (FAC).

The poor cluster of which patients were discharged showed the greatest improvement in relation to the other groups. Significant differences between this group and the other groups with reference to changes between admission- and discharge scores appeared for balance (BBS), ADL (BI), walking ability (FAC), arm function (FAT) (Kruskal- Wallis Test p < 0.01).

## Discussion

Using cluster analysis, with relevant patient characteristics (age, gender, stroke type, first stroke, localization stroke), balance, arm function, walking abilities, activities of daily living, depressive complaints and neuropsychiatric symptoms, we were able to identify two clusters of patients: those in fair/good condition on admission and those in poor condition on admission. Compared to patients in the poor cluster, patients in the good cluster performed significantly better on all assessments. Of the measurements that were used, balance (BBS) was best at separating patients into the poor and good cluster, followed by walking abilities (FAC) and ADL (BI). In this regard, our results were comparable to those of other studies of predictors of functional outcome. Although there is controversy in the field of stroke research regarding predictors of stroke outcome, in most studies age and disability have a stronger association with negative outcome than neuropsychiatric symptoms and depressive complaints [8,10,31]. Interestingly, in our sample, neuropsychiatric symptoms and depressive complaints were significant factors to separate patients into the poor and good cluster, and age was not a significant factor.

In the poor cluster, score changes were more pronounced than in the good cluster. This indicates that patients in poor condition on admission had a greater chance of further improvement. Within each cluster, a group of patients was discharged to an independent/assisted-living situation and a group was referred to a nursing home for long-term care. Nevertheless, patients in poor condition on admission had a higher risk of being referred to a nursing home for long-term care, although remarkably, half of the patients were discharged to an independent/assisted-living situation. This is in agreement with the findings in previous studies that discharge to an independent/assisted-living situation appears to be difficult to predict on the basis of on admission data for patients in poor condition upon admission. Predictions about discharge can be misleading if therapists and clinicians only take initial functional status as a the basis for discharge [32], since they risk overlooking patients who go on to regain enough functionality to be discharged to an independent/assisted-living situation. Rehabilitation programs that provide algorithms for multidisciplinary collaboration and evaluation on the basis of continuous monitoring of the physical and psychological condition of patients can be helpful in providing optimal individually tailored rehabilitation care [33,34].

Patients in the poor cluster who were discharged to an independent/assisted-living situation had, in general, the same discharge scores as patients in the good cluster on admission. Discharged patients in the poor cluster improved more than discharged patients in the good cluster. In this study, the overall percentage of patients who were discharged to an independent/assisted-living situation approached 70%. To increase this percentage, stroke specific rehabilitation programs can be implemented. These may be effective in improving functional performance [14,35], and need to incorporate high intensity therapy for patients in poor condition. Strikingly, although it has been shown that patients with a poor prognosis benefit more from higher-intensity therapy than patients who are in good condition on admission [36], there is some evidence that patients with severe stroke receive less therapy than patients with mild stroke [13]. We hypothesize that a more protocolized, comprehensive and intensive multidisciplinary rehabilitation for patients in poor condition on admission may have a positive effect on rehabilitation outcomes and, as a result, the percentage of patients who can be discharged to an independent/assisted-living situation may increase.

Neuropsychiatric symptoms and depressive complaints were significant factors to separate patients into the poor and the good cluster. Rehabilitation programs should, next to balance and functional status, also address neuropsychiatric symptoms and depressive complaints, which may increase during rehabilitation [15]. In addition, rehabilitation programs should define roles for the entire multidisciplinary team, including nursing staff on the rehabilitation ward. For a more comprehensive and intensive rehabilitation program, a therapeutic climate is needed, and nurses are rehabilitators par excellence because of their continuous presence on the rehabilitation ward [37]. It is important that nurses encourage patients to perform simple exercises, such as reaching for objects and rising from a chair. They should also walk with patients and support them in as many meaningful activities during daily life as possible. Nurses need to determine which activities are therapeutic and contribute positively to rehabilitation. This may lead to an increase of discharge-rates specifically for patients in the poor cluster.

We observed only modest improvements in the patients in the good cluster, raising the question whether these patients might have been be better off undergoing rehabilitation in the community or in day-care rehabilitation center rather than in an institution. Directors can organize stroke rehabilitation in a home environment by implementing an ambulatory operating “expert stroke team” comprising multidisciplinary team members from the SNF (including an elderly care physician [5]). Rehabilitation in the homes of patients or in a day-care center would not only be beneficial to patients but is also more cost-effective. Costs of outpatient rehabilitation are less than the costs of an admission to a Dutch SNF: the average costs per person per year are 95.000 euros for institutional SNF care (inclusive intensified therapies) and 5.200 euros for home care (exclusive 65 euros per hour for intensified therapies). Consequently, home-care or day-care could decrease health care costs [38,39].

A limitation of our research is the risk of selection bias due to missing data from patients. However, for all variables except for age, the mean results on admission were not significantly different for patients with incomplete data versus the patients with complete data. Therefore, we believe that our results are applicable to the majority of patients who are admitted to SNFs for rehabilitation.

Research in geriatric rehabilitation is scarce, specifically in those patients who are in poor condition. Therefore, further research is required to identify factors that may contribute to improvement in patients in poor condition upon admission, as well as factors associated with declining scores, which may precede the unsuccessful rehabilitation of patients in good condition on admission. In addition, it is recommended to conduct an intervention-study to investigate therapy-intensity in patients in poor condition. Lastly, there is a need to investigate whether patients can successfully undergo rehabilitation in their home or in a day-care setting to avoid admission to a SNF, and to explore the cost-effectiveness of organizing geriatric rehabilitation in/from the SNF. The results of such studies will provide more insight into the complex circumstances facing geriatric patients with stroke.

## Conclusions

Through cluster analysis, two clusters of patients were identified: patients in fair/good condition on admission and patients in poor condition on admission. Patients in poor condition on admission were more likely to be referred to a facility for long-term care, but this was certainly not the case for all patients. Almost half of these patients were discharged to an independent or assisted living situation, which implied that also in patients in poor condition on admission, discharge to an independent or assisted living situation was an attainable goal. It is important to put substantial effort into the rehabilitation of patients in poor condition at admission. SNFs can develop specific rehabilitation programs for patients in both poor and good condition on admission in order to offer tailored care and support.

## Competing interests

The authors declare that they have no financial or personal competing interests.

## Authors’ contributions

BB and MS are the primary investigators of the GRAMPS study; they designed the study and collected the data. The collected data was analyzed by BB and she wrote the manuscript. MS and DG participated in writing the manuscript. SZ, RK and HB participated in designing the study, analyzing the data and writing the manuscript. All authors read and approved the final manuscript.

## Funding

This study was funded by two care organizations: “Stichting De Zorgboog” and “Stichting Voor Regionale Zorgverlening (SVRZ).” They employed the primary investigators during the study period. A grant of 25,000 euros for the GRAMPS study was received from the science promotion foundation for nursing homes (SWBV). The organizations that sponsored the GRAMPS study were not involved in the design of the study, data collection, data processing, or manuscript preparation.

## Pre-publication history

The pre-publication history for this paper can be accessed here:

http://www.biomedcentral.com/1472-6963/12/443/prepub
